# Cost-effective larval diet mixtures for mass rearing of *Anopheles arabiensis* Patton (Diptera: Culicidae)

**DOI:** 10.1186/s13071-017-2552-3

**Published:** 2017-12-22

**Authors:** Nanwintoum Séverin Bimbilé Somda, Kounbobr Roch Dabiré, Hamidou Maiga, Hanano Yamada, Wadaka Mamai, Olivier Gnankiné, Abdoulaye Diabaté, Antoine Sanon, Jeremy Bouyer, Jeremie Lionel Gilles

**Affiliations:** 1 Insect Pest Control Laboratory, International Atomic Energy Agency, Joint FAO/IAEA Division of Nuclear Techniques in Food and Agriculture, Vienna, Austria; 2 Institut de Recherche en Sciences de la Santé/Direction Régionale de l’Ouest, Bobo-Dioulasso, Burkina Faso; 3 Laboratoire d’Entomologie Fondamentale et Appliquée, Université Ouaga 1 Joseph Ki-Zerbo, Ouagadougou, Burkina Faso; 4 Institut de Recherche Agricole pour le Développement, Yaoundé, Cameroon

**Keywords:** Larval diet, *Anopheles arabiensis*, Tuna meal, Bovine liver powder, Brewer’s yeast, Chickpea, SIT biological control

## Abstract

**Background:**

Larval nutrition, particularly diet quality, is a key driver in providing sufficient numbers of high quality mosquitoes for biological control strategies such as the sterile insect technique. The diet currently available to mass rear *Anopheles arabiensis*, referred here to as the “IAEA diet”, is facing high costs and difficulties concerning the availability of the bovine liver powder component. To promote more affordable and sustainable mosquito production, the present study aimed to find alternative diet mixtures. Eight cheaper diet mixtures comprised of varying proportions of tuna meal (TM), bovine liver powder (BLP), brewer’s yeast (BY), and chickpea (CP) were developed and evaluated through a step by step assessment on *An. arabiensis* larvae and adult life history traits, in comparison to the IAEA diet which served as a basis and standard.

**Results:**

Four mixtures were found to be effective regarding larval survival to pupation and to emergence, egg productivity, adult body size and longevity. These results suggest that these different diet mixtures have a similar nutritional value that support the optimal development of *An. arabiensis* larvae and enhance adult biological quality and production efficiency, and thus could be used for mass rearing.

**Conclusions:**

Our study demonstrated that four different diet mixtures, 40 to 92% cheaper than the IAEA diet, can result in a positive assessment of the mosquitoes’ life history traits, indicating that this mosquito species can be effectively mass reared with a significant reduction in costs. The mixture comprised of TM + BY + CP is the preferred choice as it does not include BLP and thus reduces the cost by 92% compared to the IAEA diet.

## Background

Malaria remains a major public health burden, especially in sub-Saharan Africa where 88% of all reported cases and 90% of malaria related deaths occurred in 2015 [1]. *Anopheles arabiensis* is one of the main vectors in Africa. This species is known to have a wide distribution by adapting to various ecological zones such as desert savannah, forested areas [2] and urban areas as found in Burkina Faso [3, 4]. Described as a zoophilic, exophagic and exophilic species, in comparison to *An. gambiae* [5], *An. arabiensis* displays genetic plasticity and adapts its feeding and resting behaviours following the biotope conditions [6, 7]. This plasticity, coupled with resistance mechanisms [4, 8] allows it to avoid insecticide-based control methods, including the use of long-lasting insecticide-treated nets and indoor residual spraying [6, 8, 9] which are the main methods that are currently being relied upon for malaria control. To better control this species, biological control tactics, including the sterile insect technique (SIT), appear to be promising alternatives [10].

SIT is based on a continuous release of laboratory-reared, sterile males into a target area, where they compete with wild males to mate with wild females, resulting in sterile mating. One recommendation to control *An. arabiensis* in Sudan entails the release of 1 million sterile males per day [11]. This requires a sufficient number of efficient males; high quality yet cost effective mass rearing is therefore mandatory. Mosquito larvae ingest nutrients not only to complete their development but also to accumulate reserves for adulthood [12]. Indeed, the innate qualities of the mosquito, including size, flight and mating capabilities, fecundity, and longevity are known to largely depend on the conditions throughout the immature, i.e. early developmental stages, particularly on the quality and amount of available larval food [13–19]. Larval diet is, therefore, one of the key drivers in mass release techniques.

Mosquitoes require proteins or amino acids, fatty acids, nucleic acids, sterols and vitamins for normal or optimal larval development to adulthood [20]. In the natural environment, food sources of *Anopheles* species include microorganisms and detritus [21]. For laboratory rearing, artificial larval diets are a mix of ingredients from animals, plants and microorganisms. Among such ingredients, tuna meal, bovine liver powder, soy meal, chickpeas, brewer’s yeast are known to be effective. However, their effectiveness relies upon their proportions within the diets and is dependent upon the mosquito species [22–25]. Mosquito mass rearing requires cost-effective production and diet ingredients are the most costly components of the process. Therefore, mass production would become more affordable and suitable, if larval feeding can be minimized while ensuring that the quality of insects produced remains high. In order to establish optimal *An. arabiensis* larval diets, the present study assesses different diet combinations, including more globally accessible ingredients, i.e. tuna meal (TM), bovine liver powder (BLP), chickpeas (CP) and brewer’s yeast (BY), on larvae and adult life history traits. Taking into account the accessibility of the ingredients, the diet mixtures were aimed at minimizing the proportion of BLP, the most expensive component, while maximizing the amount of TM and integrating CP and BY. Initially, eight mixtures were created and assessed on *An. arabiensis* larval developmental parameters first without vitamin mix (VM) (Vanderzant vitamin) as an additive and secondly with the additive. Finally, four more promising mixtures were selected and evaluated on both larval and adults life history traits.

## Methods

### Mosquito strain

The *An. arabiensis* strain used for all experiments was acquired from Dongola in the Northern state of Sudan in 2005 and has since been maintained at the Insect Pest Control Laboratory (IPCL) of the Joint FAO/IAEA Division of Nuclear Techniques in Food and Agriculture, Seibersdorf, Austria. A mixture (10 g/l) of 50% of TM, 50% BLP plus VM (4.6 g/l) [23] was used for larval feeding for routine colony maintenance. This mixture was used as a control in the present study. Larvae and adults were routinely reared in a climate-controlled room at 27 ± 1 °C, 70 ± 10% relative humidity, 12:12 h light: dark, including 1 h dusk and 1 h dawn. Adults were loaded into 30 × 30 × 30 cm cages (BugDorm-1H; MegaView, Taichung, Taiwan) with constant access to a 5% sugar solution. Defrosted bovine blood meals were provided to females for egg production.

### Diet ingredients, suppliers and costs

The supplier and the cost of each diet ingredient used in this study are shown in Table 1. The prices are estimated per 100 kg to take into account potential price reductions in the case of bulk orders expected in mass rearing facilities.

**Table 1:** Diet ingredient price per 100 kg and supplier

Ingredient	Code	Supplier	Price of 100 kg (USD)	Cost ratio compared with BLP	Proportion (*w*/w) in the IAEA diet (%)	Account in the IAEA diet cost (%)
Bovine liver powder	BLP	MP Biomedicals, Solon, OH	6300	1.00	50	98.75
Tuna meal	TM	T.C. Union Agrotech	80	78.75	50	1.25
Breyer’s yeast	BY	MP Biomedicals	1000	6.30	0	0
Chickpea flour	CP	TRS Asian’s fitness foods	328.26	19.19	0	0
Vanderzant vitamin	VM	BioServ	2800	2.25	Additive	–

### Bioassays

Experiments were performed at two levels using 90 mm diameter disposable polystyrene Petri dishes and secondly, plastic trays (40 × 29 × 8 cm). Larvae were fed with a 1% (10 g/l) diet solution in all experiments.

#### Experiment 1: Evaluation of the diet mixtures without vitamin mix on *An. arabiensis* larval development

Eight mixtures (hereafter Mix 1 to Mix 8) were developed by increasing the amount of TM and reducing the amount of BLP or replacing it with cheaper and more accessible ingredients, CP and BY. Each ingredient, except vitamin mix, was ground using a planetary ball mill PM100 (Retsch® GMBH, Haan, Germany). The final particle size of the ingredient powder was between 50 and 150 μm. The effect of each diet mixture was first assessed without VM, in comparison to the control (Mix 0) using Petri dishes. The ingredient proportions in each diet mixture are shown in Table 2. Thirty-two first-instar larvae (L1) less than 4 h old were transferred into 90 mm diameter disposable polystyrene Petri dishes containing 32 ml of deionized water. Five replicates were performed for each diet mixture. In order to better assess the nutritional value of the diet mixtures on larval development, the experiment was performed in food stress conditions (low food amount/larva/day). 0.32 ml of 1% diet solution, equivalent to 1 mg/larva/day, was added daily to each Petri dish from day 0 to day 9. The Petri dishes were checked every 24 h and any pupae were collected, counted and transferred into small plastic cups for emergence. The date of emergence was recorded and the emerged mosquitoes counted. Larval development time and survival rates from L1 to pupa and to adult were also determined.

**Table 2:** Ingredient proportions (%) in each diet mixture

Ingredient/ Mixture	Mix 0	Mix 1	Mix 2	Mix 3	Mix 4	Mix 5	Mix 6	Mix 7	Mix 8
Tuna meal (TM) (%)	50	70	70	70	70	50	100	25	50
Bovine liver powder (BLP) (%)	50	30	15	15	0	20	0	25	20
Brewer’s yeast (BY) (%)	0	0	15	0	15	15	0	25	20
Chickpea (CP) (%)	0	0	0	15	15	15	0	25	10
Vitamin mix (VM) (4.6 g/l)	0	0	0	0	0	0	0	0	0
Total (100%)	100	100	100	100	100	100	100	100	100

#### Experiment 2: Evaluation of the diet mixtures supplied with vitamin mix on *An. arabiensis* larval development

The diet mixtures described in experiment 1 were supplemented with vitamin mix and tested using Petri dishes and following the same protocol. An equal amount of VM (corresponding to 4.6 g/l of the mixture solution) was added to each diet mixture. To avoid confusion with the previous diet mixtures, “Mix 0 to Mix 8” were coded as “Mix 10 to Mix 18”, respectively, in experiment 2. Larval development time and survival rates from L1 to pupa and to adult were recorded.

#### Experiment 3: Evaluation of the most promising diet mixtures on *An. arabiensis* larvae and adult life history traits

From assays described above, four promising diet mixtures were selected based on the time from L1 to pupa and the larval survival rate from L1 to adult and tested using plastic trays (40 × 29 × 8 cm). Four hundred first-instar larvae (L1) were transferred into each tray containing 1 l of deionized water. A 1% diet solution was added daily to each respective tray from day 0 (D0) until day 7 (D7) as follows: 8 ml on D0 and D1, 16 ml on D2 and D3, 32 ml on D4 and D5, and 64 ml on D6 and D7. Five replicates were performed for each diet mixture. Larval development time and survival rates from L1 to pupa and to adult were recorded. Adult longevity was evaluated from 3 replicates of each diet mixture. To evaluate this parameter, 3 standard rearing cages (30 × 30 × 30 cm) were stocked with 50 males and 50 females aged 1 day from the same cohort. Mortality was checked every 2 days until all of the mosquitoes were dead. Dead mosquitoes were removed and counted by sex. Fecundity was also assessed on the same mosquitoes whose longevity was followed. This allowed the sample size for each parameter to be increased, whilst considering pupae produced on the same day and evaluating the potential longevity of the mosquitoes taking into account reproduction cost including mating, blood-feeding and egg production. Defrosted cattle blood was provided to females on the third and the fourth day post-emergence, using the blood-feeding system described by Damiens et al. [26]. Oviposition cups were placed inside the cages on the sixth and the seventh day and eggs were collected on the seventh and the eighth day. Pictures of the egg papers were captured and eggs were counted using Microsoft Paint 2013. Wing length (left wing) was considered as an indicator of adult body size; measurements were taken from 10 males and 10 females randomly selected from each of the 5 replicates of each treatment.

### Statistical analysis

The R Software version 3.2.5 [27] was used to perform all statistical analyses and to make the graphs. We used binomial generalized linear mixed models fit by maximum likelihood (Laplace approximation) with the larval survival rates to pupa and to adult from the initial number of L1 as response variables, diet mixture as fix effects and the replicate as a random effect. We used a Gaussian linear mixed-effects model with the larval development times from L1 to pupation and to emergence assigned as response variables, the diet mixture as fix effect and the repeats as random effects. The survival of adult mosquitoes reared on the different diets mixtures was analyzed using Kaplan-Meier survival curves. Survival curves were compared using the coxph model where the diet mixture is the explanatory variable and survival rate is the response variable.

## Results

### Experiment 1: Evaluation of the diet mixtures without vitamin mix on *An. arabiensis* larval development

**Fig. 1:**
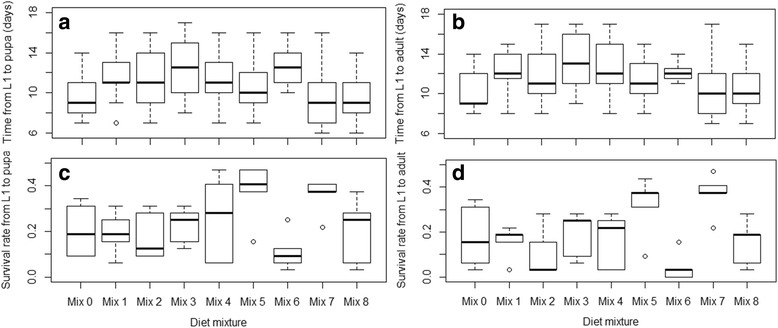
Time to pupation (**a**) and emergence (**b**) and survival rates to pupa (**c**) and to adult (**d**) using diets without vitamin mix

All of the diet mixtures were tested in food stress conditions (defined as the allotment of larval diet amounts less than the daily required amount per larvae) resulting in variable larval development times and survival rates (Fig. 1a, b). Overall, both larval survival rates to pupa and to adult were less than 50% in all treatments, including the IAEA diet (Fig. 1c, d). Larval survival rates to pupa ranged between 10 and 39% and emergence rates between 4 and 37%. The lowest rates were observed with the mixture containing only tuna meal (Mix 6) and the highest rates were observed with the mixture made of an equal amount of each of the four ingredients (Mix 7). The generalized linear mixed model with Mix 0 as the control level revealed different effects of the diets on mosquito life history traits as summarized in Tables 3 and 4. Indeed, for both larval survival rates to pupa and to adult, only Mix 5 and Mix 7 lead to significantly higher rates (*P* < 0.05) and Mix 6 reduced these rates of development (*P* < 0.05). The time from L1 to both pupa and adult was longer when using Mix 1 to Mix 5 (*P* < 0.05) and was similar between Mix 7 and Mix 8 (*P* > 0.05). Considering the slopes in the larval development time from L1 to pupation, the control, although different, was closer by order to Mix 5, Mix 3, Mix 2 and Mix 1. Mix 7, which was similar to the control in the larval development time and had a higher larval survival rate, was the most promising overall, followed by Mix 8 which was similar to the control in all parameters. Mix 6, which prolonged time to pupa (*P* < 0.05) and led to the lowest (*P* < 0.05) larval survival rate, was the least optimal diet. Hence, taking larval development time and survival rate into account, the tested diet mixtures could be classified by order of most to least promising as Mix 7, Mix 8, Mix 5, Mix 4, Mix 3, Mix 2, Mix 1 and Mix 6.

**Table 3:** Effects of diets mixtures without vitamin mix on *An. arabiensis* larval development times

		Value	SE	*df*	*t*-value	*P*-value
Time from L1 to pupa	(Intercept)	9.3961	0.4258	328	22.0672	< 0.0001***
	Mix 1	2.2485	0.6472	328	3.4742	0.0006***
	Mix 2	2.1917	0.5891	328	3.7206	0.0002***
	Mix 3	3.2251	0.5561	328	5.7978	< 0.0001***
	Mix 4	1.9290	0.5337	328	3.6143	0.0003***
	Mix 5	1.1373	0.5020	328	2.2658	0.0241**
	Mix 6	3.1756	0.6707	328	4.7351	< 0.0001***
	Mix 7	-0.2271	0.5006	328	-0.4537	0.6504
	Mix 8	0.1649	0.5370	328	0.3071	0.7590
Time from L1 to adult	(Intercept)	10.1577	0.4283	266	23.7155	< 0.0001***
	Mix 1	2.1884	0.6496	266	3.3691	0.0009***
	Mix 2	1.9216	0.6740	266	2.8509	0.0047**
	Mix 3	3.0929	0.5684	266	5.4417	< 0.0001***
	Mix 4	2.3120	0.5785	266	3.9968	0.0001***
	Mix 5	1.3320	0.4974	266	2.6780	0.0079**
	Mix 6	1.7262	0.9162	266	1.8841	0.0606
	Mix 7	-0.0946	0.4934	266	-0.1917	0.8481
	Mix 8	0.6032	0.5495	266	1.0977	0.2733

*Abbreviations*: *SE* standard error, *df* degrees of freedom

**P < 0.01, ***P < 0.001

**Table 4:** Effects of diets mixtures without vitamin mix on *An. arabiensis* larval survival rates

		Estimate	SE	*z*-value	Pr (>|z|)
Survival rate from L1 to pupa	(Intercept)	-1.3704	0.2330	-5.881	4.09e-09***
	Mix 1	-0.0790	0.2810	-0.281	0.7784
	Mix 2	-0.1621	0.2847	-0.569	0.5692
	Mix 3	0.1123	0.2736	0.411	0.6813
	Mix 4	0.2859	0.2680	1.067	0.2859
	Mix 5	0.8497	0.2564	3.314	0.0009***
	Mix 6	-0.7248	0.3186	-2.275	0.0229*
	Mix 7	0.8768	0.2561	3.424	0.0006***
	Mix 8	-0.0391	0.2794	-0.140	0.8887
Survival rate from L1 to adult	(Intercept)	-1.4992	0.2330	-6.435	1.23e-10***
	Mix 1	-0.1568	0.3010	-0.521	0.6025
	Mix 2	-0.5621	0.3306	-1.700	0.0891
	Mix 3	0.0646	0.2903	0.222	0.8240
	Mix 4	-0.0328	0.2997	-0.110	0.9128
	Mix 5	0.8163	0.2695	3.029	0.0024**
	Mix 6	-1.5293	0.4387	-3.486	0.0005***
	Mix 7	0.9769	0.26475	3.690	0.0002***
	Mix 8	-0.1893	0.3039	-0.623	0.5334

*Abbreviations*: *SE* standard error, *df* degrees of freedom

*P < 0.05, **P < 0.01, ***P < 0.001

### Experiment 2: Evaluation of the diet mixtures supplied with vitamin mix on *An. arabiensis* larval development

**Fig. 2:**
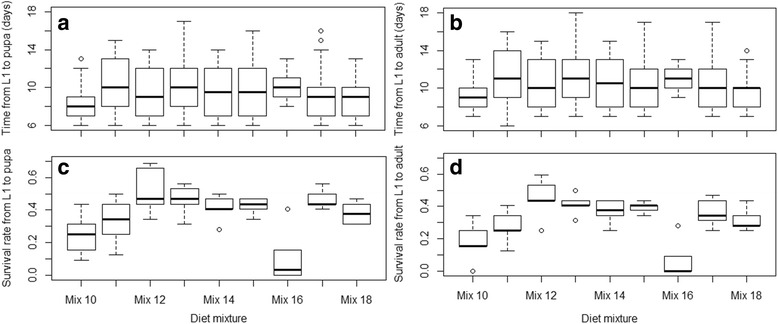
Time to pupation (**a**) and emergence (**b**) and survival rates to pupa (**c**) and to adult (**d**) using diets with vitamin mix

The larval developmental parameters using diets supplemented with vitamin mix are shown in Fig. 2a-d. In all treatments, supplementation with vitamin mix added positive effects on all parameters**.** The highest larval survival rates to pupa and to adult were observed with Mix 12. The shortest development times from L1 to pupa and to adult were observed with the control (Mix 10) and the longest with Mix 11. Differences between the diets on mosquito life history traits were observed from the generalized linear mixed model with Mix 10 (control) used as a reference level (Tables 5 and 6). The control showed a shorter time from L1 to pupa and to adult than Mix 11 to Mix 16 (*P* < 0.05) but was similar to Mix 18 (*P* > 0.05). Mix 17 resulted in a similar time to pupation as the control (*P* = 0.0538) but a longer time to emergence (*P* = 0.0109). Based on the slope values in time to pupa, the tested diets could be ranged by increasing value as by order of effectiveness as Mix 18, Mix 17, Mix 12, Mix 15, Mix 14, Mix 16, Mix 13 and Mix 11. In comparison to the control, only Mix 16 (100% tuna meal + VM) resulted in a lower larval survival rates to pupa and adult (*P* < 0.05) and Mix 11 showed similar effects (*P* > 0.05). Mix 12, Mix 13, Mix 14, Mix 15, Mix 17 and Mix 18 resulted in higher larval survival rates (*P* < 0.05). Considering the slopes in the larval survival rates to adult and in the times to pupation, the five most promising mixtures are listed respectively by decreasing value of time to pupation as Mix 12, Mix 13, Mix 15, Mix 14 and Mix 17 and by increasing value of survival rate as Mix 18, Mix 17, Mix 15, Mix 12, and Mix 14. Taking both into account, Mix 17, Mix 15, Mix 12, Mix 14 were selected and further assessed on a larger scale using plastics trays (40 × 29 × 8 cm).

**Table 5:** Effects of diets mixtures supplemented with vitamin mix on *An. arabiensis* larval development times

		Value	SE	*df*	*t-*value	*P*-value
Time from L1 to pupa	(Intercept)	8.3627	0.3903	524	21.4270	< 0.0001***
	Mix 11	2.1212	0.4975	524	4.2636	< 0.0001***
	Mix 12	1.1275	0.4566	524	2.4697	0.0138*
	Mix 13	1.7774	0.4650	524	3.8225	0.0001 ***
	Mix 14	1.3036	0.4746	524	2.7469	0.0062**
	Mix 15	1.2633	0.4710	524	2.6818	0.0076**
	Mix 16	1.7277	0.6634	524	2.6043	0.0095**
	Mix 17	0.8956	0.4634	524	1.9328	0.0538
	Mix 18	0.5323	0.4801	524	1.1086	0.2381
Time from L1 to adult	(Intercept)	8.9646	0.4332	439	20.6940	< 0.0001***
	Mix 11	2.1465	0.5579	439	3.8472	0.0001***
	Mix 12	1.4002	0.5091	439	2.7501	0.0062**
	Mix 13	2.0184	0.5164	439	3.908216	0.0001***
	Mix 14	1.5910	0.5284	439	3.010714	0.0280*
	Mix 15	1.3174	0.5198	439	2.534521	0.0116*
	Mix 16	1.8924	0.8177	439	2.314426	0.0211*
	Mix 17	1.3656	0.5343	439	2.556125	0.0109*
	Mix 18	0.7888	0.5394	439	1.462174	0.1444

*Abbreviations*: *SE* standard error, *df* degrees of freedom

*P < 0.05, **P < 0.01, ***P < 0.001

**Table 6:** Effects of diets mixtures supplemented with vitamin mix on *An. arabiensis* larval survival rates

		Estimate	SE	*z-*value	Pr (>|z|)
Survival rate from L1 to pupa	(Intercept)	-1.0986	0.1826	-6.017	1.77e-09***
	Mix 11	0.3961	0.2481	1.597	0.1104
	Mix 12	1.1736	0.2416	4.858	1.19e-06***
	Mix 13	0.9483	0.2418	3.922	8.79e-05***
	Mix 14	0.7450	0.2432	3.064	0.0022**
	Mix 15	0.7963	0.2427	3.281	0.0010**
	Mix 16	-0.9057	0.3051	-2.969	0.0030**
	Mix 17	0.9734	0.2417	4.027	5.65e-05***
	Mix 18	0.6144	0.2446	2.512	0.0120*
Survival rate from L1 to adult	(Intercept)	-1.4202	0.2069	-6.863	6.72e-12***
	Mix 11	0.5316	0.2737	1.942	0.0521
	Mix 12	1.3531	0.2640	5.125	2.97e-07***
	Mix 13	1.1555	0.2638	4.380	1.19e-05***
	Mix 14	0.9544	0.2653	3.597	0.0003***
	Mix 15	1.0415	0.2637	3.949	7.84e-05***
	Mix 16	-1.0437	0.3650	-2.859	0.0042**
	Mix 17	1.0380	0.2680	3.873	0.0001***
	Mix 18	0.7569	0.2693	2.811	0.0049 **

*Abbreviations*: *SE* standard error, *df* degrees of freedom

*P < 0.05, **P < 0.01, ***P < 0.001

### Experiment 3: Evaluation of promising diet mixtures on *An. arabiensis* larval and adult life history traits

Four diet mixtures were selected and tested at larger scale using plastic trays (40 × 29 × 8 cm): Mix 12:70% TM + 15% BLP + 15% BY + VM; Mix 14: 70% TM + 15% BY +15% CP + VM; Mix 15: 50% TM + 20% BLP + 15% BY +15% CP + VM; Mix 17: 25% TM + 25% BLP + 25% BY +25% CP + VM.

#### Effect of four selected diets on *An. arabiensis* larval development and adult body size, egg production and survival

**Fig. 3:**
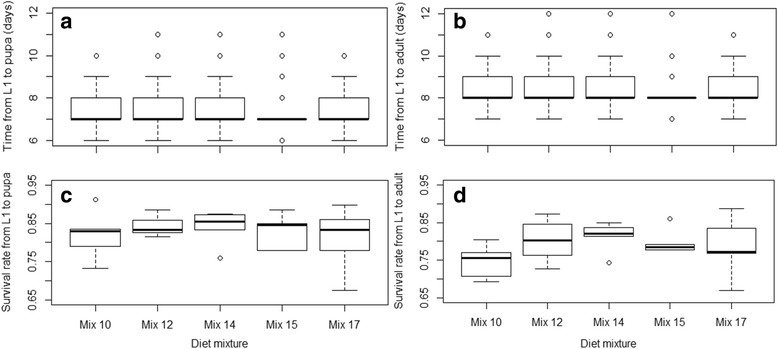
Time to pupation (**a**) and emergence (**b**) and survival rates to pupa (**c**) and to adult (**d**) using promising diet mixtures: Mix 12, Mix 14, Mix 15 and Mix 17

**Fig. 4:**
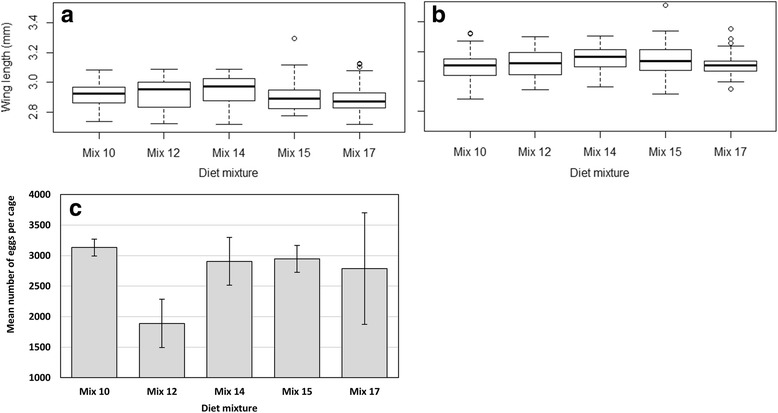
*Anopheles arabiensis* male (**a**) and female (**b**) wing length, egg production (**c**) using promising diets mixtures: Mix 12, Mix 14, Mix 15 and Mix 17

The larval and adult developmental parameters resulting from each diet mixture and the proportion of price reduction in relation to the control are summarized in Fig. 3a-d. These diet mixtures cost 40–92% less than the control. Overall, survival rates from L1 to pupa ranged between 75 and 86%, and survival rates from L1 to adult ranged between 70 and 83%. The assessed diet mixtures resulted in similar or better larval survival rates than the control (Table 7). Indeed, all treatments were similar to the control with respect to survival rates from L1 to pupa (*P* > 0.05) and resulted in higher survival rates from L1 to adult (*P* < 0.05), except for Mix 15 (*P* = 0.1729). A significant difference was observed in the effects of the assessed mixtures on larval development times (Table 8). Mix 14 and Mix 15 resulted in a shorter time from L1 to pupation while Mix 12 and Mix 17 were similar to the control. Regarding the time to emergence, Mix 12 and Mix 14 resulted in longer times (*P* < 0.05), Mix 15 in a shorter time (*P* < 0.0009) and time to emergence for Mix 17 was similar to the control (*P* = 0.1080). Considering the times from L1 to pupa and the survival rates from L1 to adult, Mix 14 would be the preferred choice. Adult wing length and egg production following the diet mixture are shown in Fig. 4a-c. No significant differences were observed between the treatments and the control for male as and female wing length (Table 8, *P* > 0.05), except for females from Mix 14 which were larger (Table 5, *P* = 0.0098). All of the mixtures had similar effects on egg production (Table 8, *P* > 0.05).

**Table 7:** Effects of four promising diets mixtures on *An. arabiensis* larval survival rates

		Estimate	SE	*z-*value	Pr (>|z|)
Survival rate from L1 to pupa	(Intercept)	1.5267	0.0990	15.417	< 2e-16***
	Mix 12	0.1652	0.0848	1.947	0.0515
	Mix 14	0.1351	0.0844	1.602	0.1093
	Mix 15	-0.1402	0.0808	-1.735	0.08 27
	Mix 17	-0.0732	0.0816	-0.898	0.3694
Survival rate from L1 to adult	(Intercept)	1.0821	0.0804	13.464	< 2e-16***
	Mix 12	0.3225	0.0762	4.233	2.31e-05***
	Mix 14	0.3902	0.0770	5.064	4.11e-07***
	Mix 15	0.1005	0.0737	1.363	0.1729
	Mix 17	0.2273	0.0750	3.029	0.0025**

*Abbreviations*: *SE* standard error, *df* degrees of freedom

**P < 0.01, *** *P* < 0.001

**Table 8:** Effects of four promising diets mixtures on *An. arabiensis* larval development times, adult wing length and egg production

		Value	SE	*df*	*t*-value	*P*-value
Time from L1 to pupa	(Intercept)	7.319910	0.0597	8206	122.5788	< 0.0001***
	Mix 12	0.0167	0.0187	8206	0.8935	0.3716
	Mix 14	-0.0412	0.0188	8206	-2.1969	0.0281*
	Mix 15	-0.1085	0.0190	8206	-5.7029	< 0.0001***
	Mix 17	-0.0249	0.0189	8206	-1.3141	0.1889
Time from L1 to adult	(Intercept)	8.2610	0.0607	7822	136.0896	< 0.0001***
	Mix 12	0.0499	0.0188	7822	2.6503	0.0081**
	Mix 14	0.0719	0.0188	7822	3.8224	0.0001***
	Mix 15	-0.0633	0.0191	7822	-3.3139	0.0009***
	Mix 17	0.0305	0.0189	7822	1.6075	0.1080
Male wing length	(Intercept)	2.9150	0.0242	241	120.4781	< 0.0001***
	Mix 12	0.0066	0.0161	241	0.4116	0.6810
	Mix 14	0.0281	0.0161	241	1.7438	0.0825
	Mix 15	-0.0020	0.0161	241	-0.1241	0.9013
	Mix 17	-0.0243	0.0161	241	-1.5091	0.1326
Female wing length	(Intercept)	3.1142	0.0205	241	152.2137	< 0.0001***
	Mix 12	0.0069	0.0167	241	0.4126	0.6803
	Mix 14	0.0434	0.0167	241	2.6051	0.0098**
	Mix 15	0.0277	0.0167	241	1.6629	0.0976
	Mix 17	-0.0028	0.0167	241	-0.1704	0.8648
Egg production	(Intercept)	3132.6667	492.5974	8	6.3595	0.0002***
	Mix 12	-1243.6667	546.1322	8	-2.2772	0.0523
	Mix 14	-226.3333	546.1322	8	-0.4144	0.6894
	Mix 15	-186.3333	546.1322	8	-0.3412	0.7418
	Mix 17	-346.3333	546.1322	8	-0.6342	0.5437

*Abbreviations*: *SE* standard error, *df* degrees of freedom

*P < 0.05, **P < 0.01, ***P < 0.001

#### Effects of four diets on *An*. *arabiensis* male and female survival

**Fig. 5:**
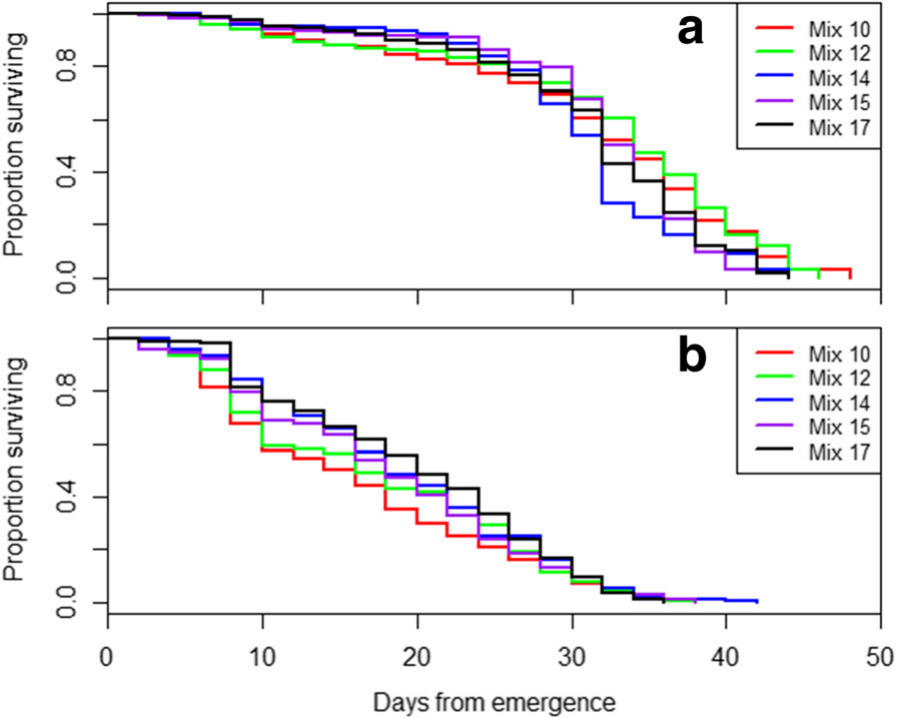
Survival curves of *An. arabiensis* males (**a**) and females (**b**) following the larval diet mixtures: Mix 12, Mix 14, Mix 15 and Mix 17

The Log-rank (Mantel-Cox) test showed different survivorships between the adults from larvae fed on the different diet mixtures for females (Log-rank test, *χ* ^2^ = 9.6, *df* = 4, *P* = 0.0483) and males (Log-rank test, χ^2^ = 26.1, *df* = 4, *P* = <0.0001). Cox proportional hazard model with Mix 10 (control) as reference showed a significant difference in the survivorship between the adults fed on the assessed diet mixtures and the control, in both sexes. For males, except for Mix 12 which had a similar effect to the control (*P* = 0.4443), Mix 14, Mix 15 and Mix 17 increased the risk of death by 1.65-, 1.36- and 1.33-fold (*P* < 0.05), respectively (Table 9). Conversely for females, Mix 14 and Mix 17 significantly reduced the hazard of death by 25 and 30% (*P* < 0.05), respectively, while Mix 12 and Mix 15 were similar to the control (*P* > 0.05) (Table 9). Overall for each treatment, males survived longer than females. The median time of survival for Mix 10 (control), Mix 12, Mix 14, Mix 15 and Mix 17, was 34, 34, 32, 33 and 32 days, respectively for males, and 15, 16, 18, 18 and 20 days, respectively for females. Over the first 30 days after emergence, the survival curves for males from the control and Mix 12 were below those of their counterparts from other mixtures (Fig. 5a). The survival curve for females from the control was the lowest followed closely by that of females from Mix 12 (Fig. 5b).

**Table 9:** Effects of four promising diets mixtures on *An. arabiensis* male and female longevity

	Diet	Coef	Exp (coef)	SE (Coef)	*z-*value	Pr (>|z|)
Males	Mix 12	-0.1031	0.9020	0.1348	-0.765	0.4443
	Mix 14	0.5026	1.6529	0.1359	3.697	0.0002***
	Mix 15	0.3088	1.3618	0.1345	2.296	0.0217*
	Mix 17	0.2872	1.3327	0.1346	2.134	0.0328*
Females	Mix 12	-0.1623	0.8502	0.1297	-1.251	0.2109
	Mix 14	-0.3281	0.7203	0.1298	-2.528	0.0115*
	Mix 15	-0.2158	0.8059	0.1296	-1.665	0.0958.
	Mix 17	-0.3500	0.7047	0.1290	-2.714	0.0067**

*Abbreviations*: *Coef* coefficient, *Exp* Exponential, *SE* standard error

*P < 0.05, **P < 0.01, ***P < 0.001

## Discussion

In this study, eight diet mixtures were designed and evaluated for *An. arabiensis* larval development in order to identify accessible and cost-effective diet ingredients for mass rearing. The trial assessing diets without any addition of vitamin mix has revealed that this species can develop on all eight diet mixtures from L1 to the adult stage. This result suggests that all diet formulations provide the required nutrients for the larval development. The difference in efficacy between treatments may rely on the quality and quantity of nutrients, which also depend on the quality and proportion of ingredients in each mixture. This is shown by the lowest effectiveness observed with the pure tuna meal diet. The addition of vitamin mix had a positive effect resulting in a higher larval survival rate and shorter development time in all treatments as previously observed by Damiens et al. [23]. The pure diet of TM, BLP, CP, or a combination of these has been reported to be effective for *Anopheles* larval development, but dependent on the amount per larva per day [23, 24]**.** The low overall larval survival rates observed (including controls), may have been due to the low quantity of diet per larva per day [28].

Following these preliminary tests, the diet mixtures Mix 12, Mix 14, Mix 15 and Mix 17 were found to be the most promising among the eight tested mixtures, taking into account the time to pupation and the larval survival rate to adult. The evaluation of these four mixtures using larger rearing trays has revealed that all allow *An. arabiensis* to complete its development from L1 to adult mosquito, with fecund females. The larval survival rates to pupa and to adult corroborate the findings of Damiens et al. [23] and Khan et al. [24] who both evaluated larval diets for *Anopheles* species. All treatments, control included, were found to have similar or better effects on most of the assessed parameters including larval survival rates, duration of immature stages, egg production and adult body size. As fecundity and mating capabilities are known to rely on adult body size [15, 17–19, 28] the similarity in the body size correlates with the lack of difference observed in the egg production and indicates a probable similarity in male mating capabilities. However, since the success of SIT requires high quality of produced males in terms of mating competitiveness, further study should be conducted to evaluate this parameter on mass-reared males using the different promising larval diets to definitely figure out the most suitable diet mixture.

Overall, males were found to survive longer than females as observed in other studies [29, 30] which addressed different objectives using the same strain as in our experiment. For all diet mixtures, males had high survival rates as more than 95% were alive after 8 days, more than 90% after 15 days, and more than 50% after 30 days. This survivorship, higher than that observed by Khan et al. [24], could be an advantage leading to maximised opportunities for mating activities in the field. Indeed, according to Sawadogo et al. [31], 4–8 days-old is optimum for male *Anopheles gambiae* (*s.s.*) to successfully inseminate females. More than 50% survival was recorded after 15 days in females from all diet mixtures. This result showed that a maximum egg production could be achieved in the mass rearing unit from females from all treatments. Indeed, the largest number of eggs has been found to be produced within the mass rearing cage during the first 15 days [32].

The similar outcomes of the assessed mixtures may be related to their similarity in the composition of nutrients that influence mosquito life history traits. Nutritional requirements of mosquito larvae are known to include amino acids, such as asparagine, arginine, glycine, histidine, isoleucine, leucine, lysine, methionine, phenylalanine, proline, serine, threonine, tryptophan and valine [20, 33, 34]**.** These elements could be provided by each ingredient because they are found in all organisms in the form of proteins [21, 35]. In addition, the VM provides all essential vitamins for optimal development such as thiamine, riboflavin, pyridoxine, nicotinic acid, calcium pantothenate, folic acid, biotin and choline [20]. Moreover, fatty acids and particularly polyunsaturated fatty acids (PUFAs), are known to play an important role in mosquito biology [36–40]. The PUFAs linoleic acid (LA, 18: 2w6), alpha-linolenic acid (ALA, 18: 3w3), arachidonic acid (AA, 20: 4w6), eicosapentaenoic acid (EPA, 20: 5w3) and docosahexaenoic acid (DHA, 22: 6w3), DGLA are essential for both larval and adult life traits as they enter into the structural composition of the cell membrane, interact with the immune system and in reproduction, and enhance survival and flight activities. The BLP, the TM and CP provide ample amounts of C_18_ and C_20–22_ PUFAs [23, 41].To develop the SIT package to control *An. arabiensis*, a standard larval diet has been developed at the Insect Pest Control Laboratory of the Joint FAO/IAEA Division of Nuclear Techniques in Food and Agriculture, composed of 50% TM (5 g/l) and 50% BLP (5 g/l) supplied with vitamin mix (4.6 g/l) as an additive [23]. Although effective in rearing high-quality adults, this diet relies on the availability of the expensive ingredient BLP, of which current and future availability are of concern. Indeed, this ingredient is more than 78-fold more costly than the TM and alone comprises about 99% of the global cost of the mixture (without any additive) (Table 1). Our study has demonstrated that four different diet mixtures that are 40–92% cheaper can result in positive mosquito life history traits, indicating that this mosquito species can be effectively mass reared with a significant reduction in cost (Table 5). The best mixtures are those which include fewer ingredients. In this context, Mix 15 and Mix 17, which contain four ingredients (TM, BLP, BY and CP), are more cumbersome to produce and more expensive than Mix 12 (TM, BLP and BY) and Mix 14 (TM, BY and CP). Moreover, taking into account the availability concern of BLP and the cost reduction in each diet mixture in relation to the control (Table 5), we recommend using Mix 14, which costs 92% less than the original diet and where BLP has been fully removed.

## Conclusions

The present study investigated eight larval diet mixtures in order to find optimal laval diets for *An. arabiensis* mass rearing. Four diet mixtures, 40–92% cheaper than the IAEA diet, resulted in positive life history traits. The mixture comprising TM + BY + CP is the preferred choice as it does not include BLP and reduces the cost by 92% compared to the IAEA diet. These findings contribute to the development of effective, affordable, and environmentally friendly techniques to reduce *An. arabiensis* populations and to thus protect the human population from both the biting nuisance of this species and the deadly disease that it transmits.

## Abbreviations

BLP: Bovine liver powder
BY: Brewer’s yeast
CP: Chickpea
FAO: Food and agriculture organization
IAEA: International atomic energy agency
IPCL: Insect pest control laboratory
Mix: Mixture
SIT: Sterile insect technique
TM: Tuna meal
VM: Vitamin mix
